# Nitrous Oxide and Oxygen

**Published:** 1899-02-25

**Authors:** 


### NITROUS OXIDE AND OXYGEN.

At the last meeting of the Royal Medical and Chirurgical Society Dr. F. W. Hewitt examined in great detail the effects produced by the administration of definite mixtures of nitrous oxide and air, and of nitrous oxide and oxygen. The results obtained were minutely described, the following being some among the interesting conclusions arrived at. When pure nitrous oxide is administered to the human subject in such a manner that no free oxygen gains admission the resulting phenomena are partly those of anæsthesia, partly those of asphyxia. All the latter may, however, be eliminated without interfering with the anæsthetic effects of the gas by administering it with certain proportions of oxygen, either pure or as atmospheric air. In estimating the respective merits of the various mixtures used as anæsthetic agents, the investigation shows that the best results were obtained with mixtures of nitrous oxide and oxygen; the next best with mixtures of nitrous oxide and air; and the worst with nitrous oxide free from air or oxygen. Air is not nearly so suitable as oxygen for eliminating the asphyxial factors of a pure nitrous oxide administration owing to the large volume of useless nitrogen which must necessarily be inhaled. There is no one mixture which will successfully anæsthetise every patient. In order to obtain the best form of anæsthesia, oxygen should be administered with nitrous oxide by means of a regulating apparatus, the percentage of the former gas being progressively increased from 2 per cent. or 3 per cent. at the commencement of administration to between 7 and 10 per cent., according to the circumstances of the case. The longer the administration lasts the greater may be the percentages of oxygen admitted. The next best results to those obtained by the use of a regulating apparatus for nitrous oxide and oxygen are to be secured by administering certain constant mixtures of those gases; those containing 5 to 7 per cent. of oxygen being the best for men, and those containing 7 to 9 per cent. for women and children. The next best results to these are to be obtained by mixtures of nitrous oxide and air, containing 14 to 18 per cent. of the latter for men, and 18 to 22 per cent. for women and children. What in any case is clear is that deep and satisfactory anæsthesia, unaccompanied by any obvious asphyxial manifestations, may be secured at ordinary pressures by mixtures of nitrous oxide and oxygen containing even as much of the latter gas as is present in our atmosphere, which would seem to give a death-blow to the asphyxial theory of nitrous oxide anæsthesia. It is quite possible, however, that some interference with oxidation may take place during the anæsthesia, although it may be in no sense its cause.

